# Prognostic Value of Post-Transplant MRD Negativity in Standard Versus High- and Ultra-High-Risk Multiple Myeloma Patients

**DOI:** 10.3390/cancers17091565

**Published:** 2025-05-04

**Authors:** Lea Jasmin Kündgen, Dilara Akhoundova, Michele Hoffmann, Myriam Legros, Inna Shaforostova, Katja Seipel, Ulrike Bacher, Thomas Pabst

**Affiliations:** 1 Department of Medical Oncology, Inselspital, University of Bern, CH-3010 Bern, Switzerland; lea.kuendgen@students.unibe.ch (L.J.K.); dilara.akhoundovasanoyan@insel.ch (D.A.); michele.hoffmann@insel.ch (M.H.); inna.shaforostova@insel.ch (I.S.); katja.seipel@unibe.ch (K.S.); 2 Department of Clinical Chemistry, Inselspital, University of Bern, CH-3010 Bern, Switzerland; myriam.legros@insel.ch; 3 Department for Biomedical Research (DBMR), University of Bern, CH-3008 Bern, Switzerland; 4 Department of Hematology, Inselspital, University of Bern, CH-3010 Bern, Switzerland; veraulrike.bacher@insel.ch; 5 Center of Laboratory Medicine (ZLM), Inselspital, University of Bern, CH-3010 Bern, Switzerland

**Keywords:** multiple myeloma, high risk, ultra-high risk, cytogenetics, minimal residual disease, autologous stem cell transplantation, progression-free survival, overall survival

## Abstract

**Background:** Cytogenetic abnormalities and the persistence of minimal residual disease (MRD) following autologous stem cell transplantation (ASCT) are two established prognostically unfavorable biomarkers in multiple myeloma (MM). Previous studies have shown that post-transplant MRD status is a powerful predictor of progression-free survival (PFS) and overall survival (OS). However, the impact of MRD remains poorly characterized in MM patients with high- or ultra-high-risk cytogenetics. **Objectives:** This study investigated the prognostic value of post-transplant MRD in standard- versus high- and ultra-high-risk MM. To this aim, we performed a retrospective analysis of 137 MM patients who underwent high-dose chemotherapy (HDCT) and ASCT at our institution between January 2019 and December 2021. Cytogenetics were assessed by fluorescence in situ hybridization. High-risk genomic alterations included del(17p), t(4;14), t(14;16), t(14;20), gain(1q), and *TP53* mutations, with two or more alterations defining the ultra-high-risk category. MRD was assessed in bone marrow aspirates post-ASCT using flow cytometry. **Results:** Eighty-two (60%) patients were categorized as being at standard risk, forty (29%) as high risk, and fifteen (11%) as ultra-high risk. Median follow-up was 47 months. MRD negativity was achieved in 76 (55%) patients. At 48 months, the overall PFS rate was 61% (72%, 50%, and 32% for the standard-, high-, and ultra-high-risk subgroups, respectively; *p* = 0.0004), while the OS rate was 85% (89%, 79%, and 80% in standard-, high-, and ultra-high-risk MM patients, respectively; *p* = 0.1494). Within the standard-risk subgroup, longer PFS was observed for patients achieving MRD negativity (*p* = 0.0172). High- and ultra-high-risk patients showed no significant differences in PFS when stratified by MRD status, possibly due to prompt progression to MRD positivity. **Conclusions:** Our results suggest that high- and ultra-high-risk MM patients might benefit from closer response monitoring, including dynamic MRD assessment. Further, high- and ultra-high-risk patients might require a more intensive peri-transplant treatment.

## Simple Summary

The impact of minimal residual disease (MRD) post-treatment consolidation with high-dose chemotherapy and autologous stem cell transplantation (ASCT) on patient outcomes in high- and ultra-high-risk multiple myeloma (MM) remains poorly characterized. We investigated this by analyzing outcomes from our single-center patient cohort and showed that MRD status post-ASCT fails to predict longer versus shorter progression-free survival in MM patients with high- or ultra-high-risk cytogenetics. Our results suggest that a single MRD assessment might be insufficient in this patient subgroup, which could benefit from a closer and dynamic MRD monitoring.

## 1. Introduction

Multiple myeloma (MM) is a malignant disease defined by the monoclonal expansion of plasma cells in the bone marrow. The incidence of MM displays geographical variations, but has increased globally since 1990 [1,2]. The integration of novel drugs into the treatment landscape of MM, including proteasome inhibitors, immunomodulatory drugs, and CD38-targeting antibodies, as well as, more recently CAR-T-cell therapy in later treatment lines, has resulted in a sustained increase in life expectancy in patients with MM [1].

The use of high-dose chemotherapy (HDCT) and autologous stem cell transplantation (ASCT) leads to significantly prolonged progression-free survival (PFS) following first-line induction therapy [3]. However, the majority of patients still relapse, and disease progression or treatment-related complications will ultimately lead to death [1].

The prognosis of MM patients has been shown to be influenced by several factors, including the characteristics of tumor cells, such as cytogenetic abnormalities and gene expression profile, in addition to extramedullary growth or lactate dehydrogenase levels [1]. There are notable differences between the risk stratification systems used, which differ not only in the serum markers included, but also in the cytogenetic genetic aberrations that are classified as high risk [4]. In accordance with the Revised International Staging System (R-ISS) by the International Myeloma Working Group (IMWG), the aberrations t(4;14), t(14;16) and del(17p) are defined as high risk [5]. The Mayo Clinic mSMART risk stratification system also considers t(14;20) and gain(1q) to be high-risk cytogenetics [6]. In recent years, the concept of ultra-high-risk MM has been introduced in cases where two or more high-risk aberrations are present. This designation, referred to as double-hit myeloma, is associated with a particularly poor prognosis in terms of progression-free survival (PFS) and overall survival (OS), when compared to that observed in single-hit multiple myeloma [6,7].

The assessment of minimal residual disease (MRD) has been demonstrated to be a powerful prognostic factor both for PFS and OS in multiple myeloma [8,9]. Several methods are used to determine MRD status, including multiparameter flow cytometry and next-generation sequencing (NGS). Regardless of the method used, various studies have indicated that the PFS of MM patients in complete remission with MRD negativity is markedly superior to that of MM patients in complete remission but with MRD positivity [10,11,12,13,14]. The impact of MRD status and depth of response on survival remains poorly defined in MM with high-risk or even ultra-high-risk cytogenetics [9]. This may be due to the fact that cytogenetic high-risk patients make up only 15–20% of the study population in large clinical trials [15].

Some studies have indicated that MRD positivity and high-risk cytogenetics are independent negative prognostic factors [16,17]. A meta-analysis illustrated that patients at standard risk with undetectable MRD have the best OS of all MM patients. Conversely, MRD-positive patients with high-risk cytogenetics demonstrate the most unfavorable prognoses [18]. In a previous study, Hu et al. observed that achieving MRD negativity also resulted in improved PFS and OS in high-risk patients [19]. Another study indicates that, despite achieving MRD negativity, PFS remains significantly inferior in high-risk patients compared to standard-risk patients [20]. Li et al. found no significant difference in PFS and OS between patients with high-risk disease who were MRD-negative and those with standard-risk disease who were MRD-positive [17]. Furthermore, Paiva et al. reported that the achievement of MRD negativity may potentially overcome high-risk cytogenetics [21]. Despite the concept of ultra-high-risk MM gaining increasing recognition in recent years, there have been only a few studies exploring the impact of MRD status on this particular group of individuals. In the MASTER phase 2 trial, transplant-eligible patients were treated with quadruplet induction therapy with daratumumab, carfilzomib, lenalidomide, and dexamethasone (Dara-KRd), followed by HDCT/ASCT, MRD-driven Dara-KRd consolidation, and lenalidomide maintenance if needed. Treatment-free monitoring was initiated in patients who had two consecutive negative MRD test results. The study demonstrated a significantly shortened PFS in those with ultra-high-risk cytogenetics compared to those with standard- or high-risk genetics [7].

The aim of this study is to investigate whether achieving MRD negativity should be the goal for all myeloma patients, or whether this aim could be waived for certain cytogenetic risk groups. A statistical analysis should help to facilitate a more comprehensive understanding of the prognostic significance of MRD negativity, considering the specific cytogenetic risk constellation. This approach may help optimize treatment goals for different patient subgroups, with the potential to enhance outcomes and avoid potential overtreatment.

## 2. Results

### 2.1. Patient Characteristics

A total of 137 patients were included in the study. The basal patient characteristics are outlined in Table 1. Of the total cohort, 82 patients (60%) were classified as being at standard risk, while 40 patients (29%) were identified as high risk, and 15 patients (11%) as ultra-high risk. The following genetic alterations were catalogued as high risk: del(17p), t(4;14), t(14;16), t(14;20), and gain(1q), as well as the presence of *TP53* mutations. The presence of two or more of these cytogenetic alterations was interpreted as ultra-high risk. Conversely, if none of the previously mentioned mutations were present, the patients were classified as having standard-risk genetics. An overview of the abnormalities detected is given in Appendix A Table A1, and a comprehensive list of the karyotype data can be found in Appendix A Table A2. The median age was 62 years. The majority of patients were male (67%) and were in stage II according to R-ISS (49%) at the time of diagnosis. A significant difference between the groups was observed in the proportion of patients who were in stage III according to R-ISS (standard risk 12% vs. high risk 33% vs. ultra-high risk 60%; *p* < 0.0001). The immunoglobulin IgG was the most prevalent (66%), while IgA was less common (17%). However, the proportion of IgA present between the groups varied significantly (standard risk 11% vs. high risk 20%, ultra-high risk 40%; *p* = 0.0177). IgM could only be detected in one patient. Significant differences between the risk groups were also found in the presence of light chains. Lambda light chains were detected most frequently in ultra-high-risk patients (standard risk 22% vs. high risk 33% vs. ultra-high risk 80%; *p* < 0.0001). In contrast, kappa light chains were identified in 76% of standard-risk, 68% of high-risk, and only 20% of ultra-high-risk patients (*p* = 0.0001). Anemia was present in 18% of patients, and was most prevalent among high- (33%) and ultra-high-risk (27%) patients, compared to 10% among patients with standard-risk cytogenetics (*p* = 0.0063).

### 2.2. Course of Treatment

All 137 patients received induction therapy prior to HDCT/ASCT. The regimens utilized are outlined in Appendix A Table A3. The most frequently used combination (86%) was bortezomib, lenalidomide, and dexamethasone (VRd). In preparation for stem cell apheresis, mobilization was typically achieved through the administration of a chemotherapeutic agent, such as vinorelbine or gemcitabine, in conjunction with G-CSF (58%). A further 30% of patients were mobilized with G-CSF alone, and 10% received the proteasome inhibitor ixazomib in addition to G-CSF. High-dose chemotherapy was mainly administered using treosulfan and melphalan (48%). Melphalan alone was utilized in 31% of cases, while a combination of bendamustine and melphalan was employed in the remaining 21%. A substantial number of patients (87%) received maintenance treatment with lenalidomide following HDCT/ASCT, while 9% of patients did not receive any maintenance therapy. The remaining patients were treated with other therapeutic agents, which are also listed in Appendix A Table A3. A total of 14 patients (10%) underwent additional ASCT, with 6% of standard-risk, 18% of high-risk, and 13% of ultra-high-risk patients receiving tandem ASCT. No significant differences were identified between the groups with respect to the therapeutic regimens used during the induction, mobilization, HDCT, and maintenance phases, or regarding the administration of tandem ASCT.

### 2.3. Response

Following induction, most patients achieved VGPR (47%), while 36% reached PR. Notably, 14% of patients were already in CR after induction. After HDCT/ASCT, 76% of patients achieved CR or better, with 42% attaining sCR. The remaining patients were in VGPR (14%) or PR (9%). One patient died on day 25 following HDCT, prior to the assessment of remission status. Among ultra-high-risk patients, 73% achieved CR or sCR, while 85% of the high-risk group and 72% of the standard-risk group reached this outcome. No significant differences were identified between the groups with regard to the stages of remission attained, including the achievement of post-transplant flow MRD negativity (determined on bone marrow day +15 to day +20 following ASCT). A total of 53% of patients classified as ultra-high-risk had an MRD-negative status after receiving HDCT/ASCT. Among high-risk patients, the MRD negativity rate was 60%, while in standard-risk patients it was 54% (*p* = 0.7910). Overall, 55% of patients were MRD-negative after HDCT/ASCT (Table 2).

### 2.4. Clinical Outcomes

The clinical outcomes are illustrated in Table 3. The median duration of follow-up was 47 months, with 46 months for standard-risk, 53 months for high-risk, and 49 months for ultra-high-risk patients.

**Figure 1:**
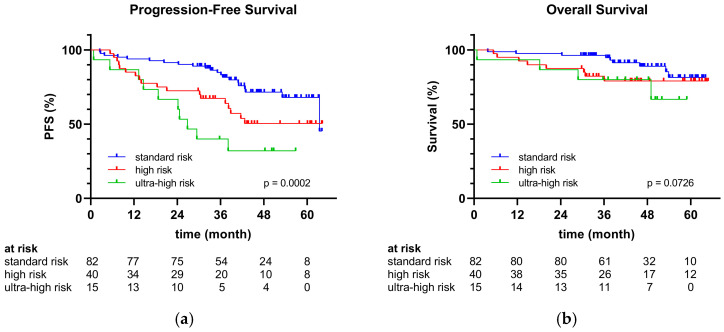
(**a**) Progression-free survival and (**b**) overall survival for standard- vs. high- vs. ultra-high-risk patients.

During the follow-up period, 44 patients experienced a relapse, representing an incidence of 32%. Patients with an ultra-high-risk or high-risk constellation exhibited a significantly higher recurrence rate than those with a standard-risk constellation (standard risk 22% vs. high risk 43% vs. ultra-high risk 60%; *p* = 0.0037). There were also significant differences in PFS (*p* = 0.0002), which is illustrated in Figure 1a. As the median PFS was not reached in all groups, the 48-month PFS rate for the overall population was 61%. PFS was significantly inferior in high-risk patients compared to standard-risk patients, and even more so in the ultra-high-risk group (48-month PFS: standard risk 72% vs. high risk 50% vs. ultra-high risk 32%; *p* = 0.0004).

A total of 21 deaths occurred (15%). No statistically significant differences in mortality rates were observed between different risk groups (*p* = 0.1868), even though—proportionally—27% of ultra-high-risk patients, 20% of high-risk patients, and 11% of standard-risk patients died. Figure 1b visualizes the OS. The overall study population demonstrated an 85% 48-month OS rate, while median OS was not reached in any group. Despite the OS rate for high- and ultra-high-risk patients being lower than for standard-risk patients, no statistically significant differences could be identified (48-month OS: standard risk 89% vs. high risk 79% vs. ultra-high risk 80%; *p* = 0.1494).

**Figure 2:**
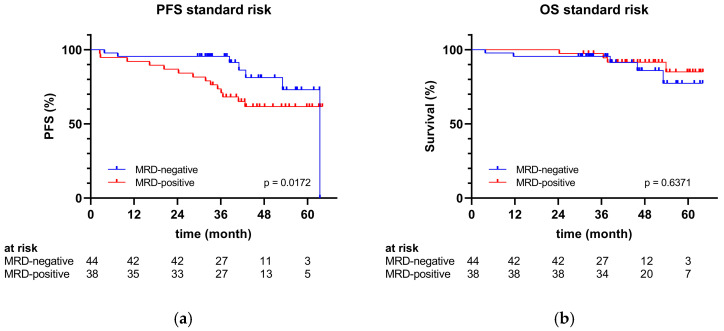
(**a**) PFS and (**b**) OS of the standard-risk group by MRD status determined by flow cytometry of bone marrow on day +15 to day +20 post-transplant.

Figure 2 illustrates the PFS and OS for patients with standard-risk cytogenetics stratified by MRD status. Within the standard-risk group, a significantly prolonged PFS was observed in patients who achieved MRD negativity compared to those who did not (*p* = 0.0172). Nevertheless, no statistically significant association was identified between OS and MRD status in patients with standard-risk genetics (*p* = 0.6371).

**Figure 3:**
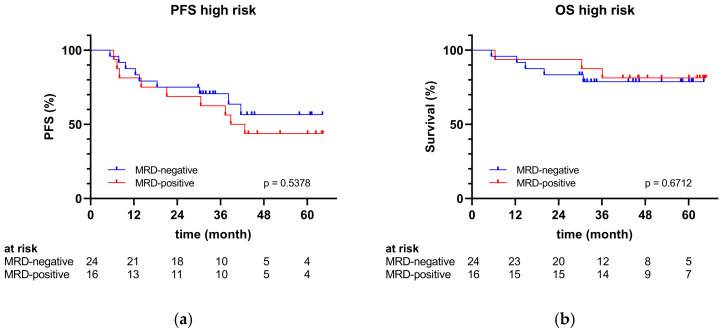
(**a**) PFS and (**b**) OS of the high-risk group by MRD status.

No significant differences were revealed within the high-risk cohort in either PFS or OS depending on MRD status post-transplant (Figure 3).

**Figure 4:**
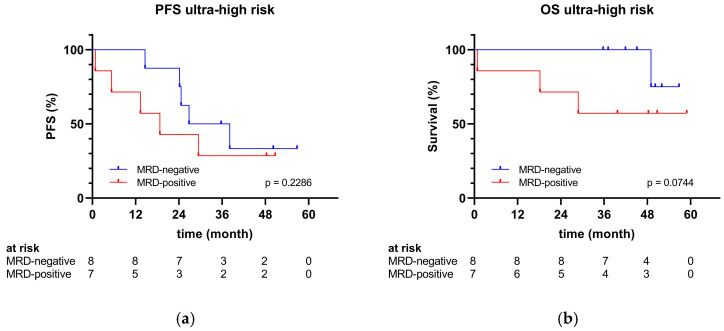
(**a**) PFS and (**b**) OS of the ultra-high-risk group by MRD status.

Likewise, no significant differences in PFS and OS were observed based on MRD status post-transplant within the ultra-high-risk group (Figure 4). However, a trend towards longer survival was observed in patients who achieved MRD negativity. Given the limited sample size of 15 individuals in the ultra-high-risk group, these findings should be cautiously interpreted.

**Figure A1:**
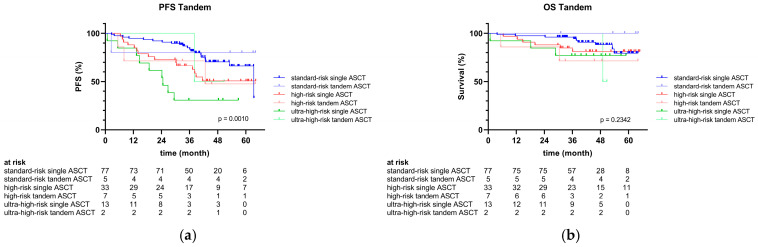
(**a**) Progression-free survival and (**b**) overall survival for standard- vs. high- vs. ultra-high-risk and single vs. tandem ASCT.

As mentioned before, 14 patients (10%) underwent tandem ASCT. The survival curves of those patients can be found in Figure A1 in the Appendix A. Due to the limited number of patients who received tandem ASCT, no further statistical analysis was performed.

## 3. Discussion

Our data suggest that MM patients with high-risk cytogenetics have shorter PFS compared to patients with standard-risk alterations, an observation that aligns with prior studies [22,23,24]. Furthermore, our findings indicate that ultra-high-risk MM is associated with an even shorter PFS. Various studies have reported similar findings [7,25]. The MASTER phase 2 trial demonstrated a markedly shortened PFS in MM patients with ultra-high-risk cytogenetics compared to those with standard- or high-risk disease [7], suggesting that even quadruplet induction therapy might be insufficient to improve outcomes for this patient subgroup. This highlights the importance of identifying novel therapeutic strategies for ultra-high-risk MM, particularly in the context of persistent MRD positivity [7].

Given the limited follow-up period of our study, our data are hypothesis-generating, and data on long-term outcomes, such as OS, are immature. In fact, median OS was not reached in any of the subgroups. In other studies, a markedly inferior OS was observed in patients with ultra-high-risk MM [7,25].

In our study, achieving MRD negativity following HDCT and ASCT determined by marrow flow cytometry in patients with standard-risk MM was associated with superior PFS. In contrast, in patients with high-risk MM, achieving MRD negativity did not appear to be sufficient to predict better outcomes. A study conducted by Chakraborty et al. demonstrated a superior PFS by reaching MRD negativity in patients with high-risk myeloma, with the exception of those with del(17p) and patients with ultra-high-risk MM [9]. A comparable conclusion was drawn by the MASTER study. The MRD-driven study design enabled patients to enter treatment-free monitoring when two consecutive negative MRD test results were obtained during the consolidation or maintenance phases. Despite similar MRD negativity rates between patients with standard-, high- and ultra-high-risk cytogenetics, patients with ultra-high-risk MM still showed a shorter PFS and a more frequent MRD resurgence after entering the treatment-free monitoring phase. As suggested by the researchers, this can be explained by the inferior outcomes of MRD-positive patients and the high progression rates of MRD-negative patients [7]. One potential explanation for patients treated at our institution is that most MM patients receive maintenance therapy with lenalidomide for a minimum of two years. A recent study showed that patients who received lenalidomide for at least two years had a longer PFS compared to those with a shorter maintenance treatment [26]. This may be sufficient for MRD-negative standard-risk MM patients, but may not be intensive enough for high-risk or ultra-high-risk patients. An analysis of patients enrolled in the Myeloma XI trial showed a significant benefit in PFS for patients with isolated del(1p), del(17p), or t(4;14) who received lenalidomide maintenance compared to the observation group. Conversely, the findings indicated a limited benefit of lenalidomide maintenance in patients with isolated gain(1q) or ultra-high-risk MM [27]. Shah et al. have suggested that ultra-high-risk patients are unlikely to benefit from lenalidomide as a single-agent maintenance therapy [28]. These patients require more intensive maintenance regimens. Multiple studies evaluated the role of daratumumab in induction and/or maintenance therapy [29,30,31,32,33]. The multicenter OPTIMUM (MUKnine) phase II trial showed that daratumumab, low-dose cyclophosphamide, lenalidomide, bortezomib, and dexamethasone (Dara-CVRd) induction with post-ASCT daratumumab, lenalidomide, bortezomib and dexamethasone (Dara-VRd) consolidation markedly enhances PFS for ultra-high-risk MM patients compared to conventional therapy [31]. The phase 3 PERSEUS trial evaluated the treatment efficacy of Dara-VRd induction and consolidation followed by Dara-lenalidomide (Dara-R) maintenance compared to VRd induction and consolidation followed by lenalidomide (R) maintenance alone in transplant-eligible patients with multiple myeloma. The risk of disease progression and death was significantly lower, while the rates of CR and MRD negativity were significantly increased in the D-VRd treatment group. The randomization process was stratified according to several factors, including cytogenetic risk [29]. A post hoc analysis showed that the combination of D-VRd followed by Dara-R maintenance is more effective than VRd followed by R maintenance in terms of PFS for all cytogenetic risk groups. However, this superiority does not appear to be significant for ultra-high-risk patients and those with isolated gain1q21 [32]. As no re-randomization prior to maintenance therapy was conducted, it is not possible to draw conclusions regarding the extent to which Dara-lenalidomide as a maintenance strategy offers an advantage over lenalidomide alone, regardless of induction [29]. However, possible negative effects of daratumumab during the induction of stem cell mobilization and engraftment in ASCT have been shown [33]. Insights relevant to the efficacy of daratumumab in maintenance therapy are offered by the phase 3 AURIGA study. The trial investigated the efficacy of Dara-R versus R maintenance in transplant-eligible and anti-CD38 naive MM patients. Data from this trial demonstrated a higher MRD-negative conversion rate within 12 months and a longer PFS for patients who received Dara-R as a maintenance strategy compared to R alone. This finding was consistent across all investigated patient groups, including those with high-risk disease [30]. However, the effect on ultra-high-risk MM patients was not examined further.

As only a small proportion of our patients underwent tandem ASCT, a conclusion regarding the effects on outcome cannot be drawn. A retrospective study conducted by the Spanish Myeloma Group indicates that double ASCT may partially overcome the adverse prognosis associated with high-risk genetic abnormalities in comparison to single ASCT [34]. An older analysis by Cavo et al. described a significant PFS benefit associated with double ASCT compared to single ASCT in patients with t(4;14) and/or t(14;16) and/or del(17p), as well a benefit for patients with an additional amp(1q) and/or del(1p) [35].

Some limitations of our study are the relatively small sample size, potentially impacting subgroup analyses. Furthermore, the short follow-up period limits the ability to draw conclusions regarding OS. Comparability with other studies is only possible to a limited extent due to the different aberrations defined as high-risk and ultra-high-risk cytogenetics.

In addition to cytogenetic abnormalities, other factors contribute to a less favorable prognosis. These include a high presence of circulating plasma cells or extramedullary disease at the time of diagnosis. Furthermore, gene expression profiling (GEP) has also been demonstrated to stratify patients according to their risk [15,28,36,37]. Given the multitude of factors that can influence the prognosis of MM patients, it remains an important task to identify a suitable risk stratification strategy. Dynamic MRD monitoring throughout the treatment course might improve the prognostic value of MRD assessment in MM patients. A few studies have already investigated MRD-guided treatment (de-)escalation for MM patients [7,38]. However, the final report of the MASTER trial suggests that these approaches may facilitate therapy de-escalation for standard-risk patients who respond well, but that the results may not be optimal for ultra-high-risk patients [38]. For these patients, it is essential to ascertain suitable treatment strategies, particularly for MM patients with MRD-positive high- or ultra-high-risk disease.

## 4. Materials and Methods

### 4.1. Patients

This retrospective study included patients diagnosed with MM who underwent HDCT and ASCT at the University Hospital of Bern, Switzerland, between January 2019 and December 2021. Patients were excluded if data on cytogenetics were unavailable or if MRD assessment had not been performed. The study was approved by the local ethics committee of Berne, Switzerland (approval number: 2024-01246; decision date: 7 August 2024).

### 4.2. Cytogenetics

Cytogenetics were assessed in bone marrow aspirates at the time of diagnosis using fluorescence in situ hybridization (FISH). *TP53* mutations were identified by next-generation sequencing, although this was not performed as a routine testing procedure. For the purpose of analysis, patients were assigned to one of three groups according to their genetic risk profiles based on cytogenetic abnormalities or the presence of a *TP53* mutation: the standard-risk group, the high-risk group, or the ultra-high-risk group. High-risk aberrations were defined as del(17p), t(4;14), t(14;16), t(14;20), gain(1q), and *TP53* mutations. In case of multiple high-risk aberrations, the patient was classified as ultra-high-risk. Conversely, the absence of high-risk mutations was interpreted as standard-risk genetics.

### 4.3. MRD

A bone marrow biopsy and flow cytometric immunophenotyping were performed at Bern University Hospital’s Center for Laboratory Medicine between days +15 and +20 post-ASCT to assess minimal residual disease (MRD) status. For MRD assessment, leukemia aberrant immunophenotype (LAIP)-positive cells were quantified within the white blood cell compartment. The presence of <10^−5^ aberrant plasma cells in the bone marrow was considered indicative of MRD negativity.

### 4.4. Endpoints

The primary endpoint of the study was PFS, and secondary endpoints were the correlation of PFS with risk categories and MRD status, as well as OS. PFS was defined as the time elapsed between HDCT and either a first relapse, progression, or death, irrespective of the cause of death. OS was defined as the time between HDCT and death, also regardless of the cause. The data cut-off date was 31 May 2024. In the absence of a current follow-up report, it was assumed that the patient had not experienced a relapse or had not died by the time of the analysis. Treatment responses were assessed using the IMWG response criteria [10].

### 4.5. Statistical Analysis

The data were collected and analyzed using Microsoft Excel 2016. Percentages were rounded to whole numbers. To ensure that the total percentage equaled 100%, the percentage of the largest non-rounded difference value was adjusted. The Kruskal–Wallis test, Chi-square test, and log-rank test were performed to assess comparisons between the standard-, high- and ultra-high-risk groups. Kaplan–Meier curves for PFS and OS were generated using GraphPad Prism version 8.0.1, and *p*-values were calculated using the Gehan–Breslow–Wilcoxon test.

## 5. Conclusions

Patients with high- and ultra-high-risk MM exhibited shorter PFS than patients with standard-risk disease. Moreover, while post-transplant flow MRD negativity correlated with longer PFS in standard-risk MM patients, single MRD assessments showed no correlation with PFS in high- and ultra-high-risk patients. These results suggest that high- and ultra-high risk MM patients might benefit from closer response monitoring, including dynamic MRD assessment. Further, these patients might require more intensive peri-transplant management.

## Figures and Tables

**Table 1:** Patient characteristics at initial diagnosis.

Parameter	Total	Standard-Risk Genetics	High-Risk Genetics ^a^	Ultra-High-Risk Genetics ^b^	*p*-Value
Patients, n (%)	137 (100)	82 (60)	40 (29)	15 (11)	
Median age, years (range)	62 (31–75)	61 (35–75)	62 (31–75)	63 (44–75)	0.9460
Sex, n (%)					
Male	92 (67)	58 (71)	24 (60)	10 (67)	0.4952
Female	45 (33)	24 (29)	16 (40)	5 (33)	0.4952
R-ISS, n (%)					
I	33 (24)	24 (29)	8 (20)	1 (7)	0.1314
II	67 (49)	43 (53)	19 (47)	5 (33)	0.3873
III	32 (23)	10 (12)	13 (33)	9 (60)	<0.0001
Unknown ^c^	5 (4)	5 (6)	0 (0)	0 (0)	-
Paraprotein subtype, n (%)					
Lambda light chain	43 (31)	18 (22)	13 (33)	12 (80)	<0.0001
Kappa light chain	92 (67)	62 (76)	27 (68)	3 (20)	0.0001
IgG	91 (66)	60 (73)	22 (55)	9 (60)	0.1170
IgA	23 (17)	9 (11)	8 (20)	6 (40)	0.0177
IgM	1 (1)	0 (0)	1 (2)	0 (0)	-
Light-chain-only	20 (15)	11 (13)	9 (23)	0 (0)	0.0973
Unknown	2 (1)	2 (3)	0 (0)	0 (0)	-
Hypercalcemia ^c^, n (%)	18 (13)	9 (11)	7 (18)	2 (13)	0.6055
Renal insufficiency ^d^, n (%)	15 (11)	6 (7)	6 (15)	3 (20)	0.2184
Anemia ^e^, n (%)	25 (18)	8 (10)	13 (33)	4 (27)	0.0063
Osteolytic lesions, n (%)	106 (77)	64 (78)	33 (83)	9 (60)	0.2011
Bone marrow infiltration, median, % (range)	60 (2–100)	50 (2–100)	60 (5–100)	60 (15–80)	0.1080

R-ISS: Revised International Staging System; ^a^ 1 of t(4;14), t(14;16), t(14;20), del(17p), gain(1q), or *TP53* mutation; ^b^ ≥2 of t(4;14), t(14;16), t(14;20), del(17p), gain(1q), or *TP53* mutation; ^c^ calcium > 2.75 mmol/L; ^d^ serum creatinine > 177 µmol/L; ^e^ hemoglobin < 100 g/L.

**Table 2:** Response and MRD.

Parameter	Total (*n* = 137)	Standard-Risk Genetics (*n* = 82)	High-Risk Genetics (*n* = 40)	Ultra-High-Risk Genetics (*n* = 15)	*p*-Value
Remission status after induction, n (%)					
CR	19 (14)	11 (13)	6 (15)	2 (13)	0.9701
VGPR	64 (47)	38 (46)	21 (52)	5 (33)	0.4445
PR	49 (36)	30 (37)	12 (30)	7 (47)	0.5019
SD	5 (3)	3 (4)	1 (3)	1 (7)	-
PD	0 (0)	0 (0)	0 (0)	0 (0)	-
Remission status after HDCT/ASCT ^a^, n (%)					
sCR	58 (42)	34 (41)	18 (45)	6 (40)	0.9160
CR	46 (34)	25 (31)	16 (40)	5 (33)	0.5795
VGPR	19 (14)	13 (16)	5 (12)	1 (7)	0.6112
PR	13 (9)	10 (12)	1 (3)	2 (13)	0.1987
SD	0 (0)	0 (0)	0 (0)	0 (0)	-
PD	0 (0)	0 (0)	0 (0)	0 (0)	-
MRD ^b^ status post-transplant					
MRD-negative	76 (55)	44 (54)	24 (60)	8 (53)	0.7910
MRD-positive	61 (45)	38 (46)	16 (40)	7 (47)	0.7910
MRD in patients with tandem ASCT, n (%)					
MRD-negative after first HDCT/ASCT	4 (3)	0 (0)	3 (8)	1 (7)	-
MRD-negative after second HDCT/ASCT	7 (5)	2 (2)	4 (10)	1 (7)	-
MRD-positive after second HDCT/ASCT	7 (5)	3 (4)	3 (8)	1 (7)	-

sCR: stringent complete remission; CR: complete remission; VGPR: very good partial remission; PR: partial remission; SD: stable disease; PD: progressive disease; HDCT: high-dose chemotherapy; ASCT: autologous stem cell transplantation; MRD: minimal residual disease by marrow flow cytometry at days 15–20 post-transplant; ^a^ one patient died before the remission status was assessed; ^b^ after single ASCT or tandem ASCT.

**Table 3:** Outcomes.

Parameter	Total (*n* = 137)	Standard-Risk Genetics (*n* = 82)	High-Risk Genetics (*n* = 40)	Ultra- High-Risk Genetics (*n* = 15)	*p*-Value
Best response after HDCT/ASCT, n (%)					
sCR	75 (55)	43 (52)	24 (60)	8 (53)	0.7284
CR	47 (34)	27 (33)	14 (35)	6 (40)	0.8635
never in CR	15 (11)	12 (15)	2 (5)	1 (7)	0.2374
Relapse after HDCT/ASCT, n (%)	44 (32)	18 (22)	17 (43)	9 (60)	0.0037
48-month PFS rate, %	61	72	50	32	0.0004
Death, n (%)	21 (15)	9 (11)	8 (20)	4 (27)	0.1868
Disease progression	10 (7)	3 (4)	5 (13)	2 (13)	-
HSCT-related cause	1 (1)	1 (1)	0 (0)	0 (0)	-
Other or unknown cause	10 (7)	5 (6)	3 (8)	2 (13)	-
48-month OS rate, %	85	89	79	80	0.1494
Median follow-up after HDCT/ASCT, months (range)	47 (0.8–65)	46 (4–64)	53 (5–65)	49 (0.8–59)	0.4512

## Data Availability

All primary data can be provided upon request.

## Appendix A

**Table A1:** Cytogenetics.

Parameter	Total (*n* = 137)	Standard-Risk Genetics (*n* = 82)	High-Risk Genetics (*n* = 40)	Ultra-High-Risk Genetics ^b^ (*n* = 15)
High-risk cytogenetic abnormality, n (%)	55 (40)	0 (0)	40 (100)	15 (100)
del(17p)	9 (7)	-	5 (13)	4 (27)
t(4;14)	23 (17)	-	14 (35)	9 (60)
t(14;16)	2 (1)	-	1 (3)	1 (7)
t(14;20)	1 (1)	-	0 (0)	1 (7)
+1q	31 (23)	-	17 (43)	14 (93)
*TP53* mutation	6 (4)	-	3 (8)	3 (20)
Standard-risk cytogenetic abnormality ^a^, n (%)	117 (85)	82 (100)	23 (58)	12 (80)
t(11;14)	26 (19)	20 (24)	5 (13)	1 (7)
t(6;14)	0 (0)	0 (0)	0 (0)	0 (0)
del(1p)	14 (10)	6 (7)	4 (10)	4 (27)
Hypodiploidy	27 (20)	11 (13)	9 (23)	7 (47)
−13	24 (18)	9 (11)	8 (20)	7 (47)
Hyperdiploidy	53 (39)	38 (46)	10 (25)	5 (33)
+3	34 (25)	24 (29)	7 (18)	3 (20)
+5	25 (18)	19 (23)	3 (8)	3 (20)
+7	22 (16)	15 (18)	3 (8)	4 (27)
+9	25 (18)	20 (24)	3 (8)	2 (13)
+11	22 (16)	15 (18)	3 (8)	4 (27)
+15	27 (20)	20 (24)	5 (13)	2 (13)
+19	25 (18)	20 (24)	3 (8)	2 (13)
+21	15 (16)	12 (15)	1 (3)	2 (13)

^a^ Only the most frequently identified standard-risk abnormalities are shown. The following aberrations were detected in ≤5% of cases: t(9;14), Monosomy 2, Monosomy 4, Monosomy 6, Monosomy 8, Monosomy 9, Monosomy 10, Monosomy 12, Monosomy 14, Monosomy 16, Monosomy 17, Monosomy 18, Monosomy 20, Monosomy 22, -Y, -X, del(2q), del(6q), del(8q), del(11q), del(13q), del(14q), de(15q), del(16q), del(17q), del(20q), del(5p), del(7p), del(20p), Trisomy 2, Trisomy 4, Trisomy 6, Trisomy 8, Trisomy 10, Trisomy 12, Trisomy 13, Trisomy 14, Trisomy 16, Trisomy 17, Trisomy 18, Trisomy 20, Trisomy 22, Tetrasomy 7, Tetrasomy 9, Tetrasomy 11, Tetrasomy 14, Tetrasomy 15, Tetrasomy 16, Tetrasomy 18, Tetrasomy 19, Tetrasomy 20, gain(1p), gain(4p), gain(5p), gain(11p), gain(18p), gain(20p), gain(5q), gain(8q), gain(9q), gain(10q), gain(11q), gain(14q), gain(18q), gain(20q), +Y; ^b^ 2 patients had more than 2 high-risk aberrations.

**Table A2:** Karyotype data.

Patient	Genetic Risk Classification	Karyotype in ISCN Format
1	Standard risk	54,XY,+3,+4,+7,+9,+15,+17,+19,+21,dup(8)(q),dup(11)(q)
2	Standard risk	57,XY,+3,+4,+5,+7,+9,+9,+11,+12,+15,+15,+19
3	Standard risk	?,XY,?
4	Standard risk	48,XY,+15,+19,+21,−Y
5	Standard risk	?,XX,+?
6	Standard risk	54,XY,+3,+4,+9,+11,+15,+19,+20,+21
7	Standard risk	46,XY,t(11;14)
8	Standard risk	46,XY,t(11;14),del(16)(q)
9	Standard risk	?,XX,?
10	Standard risk	46,XY,t(11;14)
11	Standard risk	49,XX,+3,+9,+15
12	Standard risk	50,XY,+5,+9,+15,+19,del(1)(p)
13	Standard risk	51,XY,+3,+5,+11,+15,+19
14	Standard risk	54,XY,+3,+5,+7,+9,+11,+15,+19,+Y,del(1)(p)
15	Standard risk	?,XX,+?
16	Standard risk	50,XX,+1,+3,+7,+9,−13,+15
17	Standard risk	53,XY,+3,+5,+7,+9,+15,−16,+19,+21,+Y
18	Standard risk	61,XY,+1,+2,+3,+4,+5,+6,+7,+9,+11,+15,+15,+17,+19,+19,+21
19	Standard risk	50,XX,+3,+9,+9,+15,dup(4)(p),dup(11)(q),dup(20)(q)
20	Standard risk	46,XY,t(11;14)
21	Standard risk	?,XY,?
22	Standard risk	46,XY,t(11;14)
23	Standard risk	44,XY,−13,−14,del(1)(p)
24	Standard risk	46,XX,t(11;14)
25	Standard risk	55,XY,+3,+5,+9,+9,+11,+15,+19,+21,+22
26	Standard risk	46,XY,t(11;14)
27	Standard risk	?,XX,?
28	Standard risk	?,XX,+?,del(1)(p)
29	Standard risk	46,XX,del(1)(p)
30	Standard risk	46,XX,t(11;14),del(16)(q)
31	Standard risk	46,XX,t(11;14)
32	Standard risk	?,XX,?
33	Standard risk	?,XY,?
34	Standard risk	46,XY,t(11;14)
35	Standard risk	46,XX,t(11;14)
36	Standard risk	49,XY,+4,+9,+11
37	Standard risk	?,XY,?
38	Standard risk	53,XY,+3,+5,+7,+9,+9,+15,+19
39	Standard risk	53,XY,+3,+5,+9,+11,+15,+19,+Y
40	Standard risk	43,XX,−13,−14,−22
41	Standard risk	?,XY,?
42	Standard risk	55,XY,+3,+5,+6,+9,+9,+15,+15,+19,+21
43	Standard risk	45,XX,−13,dup(18)(q),del(20)(p)
44	Standard risk	55,XY,+3,+5,+7,+9,+11,+15,+19,+19,+21
45	Standard risk	43,XY,t(11;14),−13,−14,−Y
46	Standard risk	?,XX,?
47	Standard risk	46,XX,t(11;14)
48	Standard risk	50,XY,+3,+7,+9,+15
49	Standard risk	49,XY,+3,+7,+9
50	Standard risk	?,XY,?
51	Standard risk	?,XY,?
52	Standard risk	?,XY,?
53	Standard risk	?,XY,?
54	Standard risk	48,XX,+5,+9,+11,−13,−16,+19,dup(1)(p),del(17)(q),dup(18)(p)
55	Standard risk	46,XY,t(11;14)
56	Standard risk	?,XY,?
57	Standard risk	48,XY,+9,+15
58	Standard risk	?,XY,?
59	Standard risk	58,XY,+2,+3,+5,+6,+7,+9,+9,+11,+15,+15,+19,+21
60	Standard risk	42,XX,−13,−14,−16,−X
61	Standard risk	?,XY,?
62	Standard risk	65,XY,+2,+3,+5,+7,+9,+9,+11,+11,+13,+14,+15,+15,+16,+18,+18,+19,+19,+20,+20
63	Standard risk	46,XY,t(11;14)
64	Standard risk	49,XY,+6,+9,+19
65	Standard risk	?,XY,+?
66	Standard risk	47,XY,+12
67	Standard risk	46,XY,+3,−13
68	Standard risk	50,XY,+14,+14,+16,+16
69	Standard risk	?,XY,?
70	Standard risk	45,XY,−13,del(13)(q)
71	Standard risk	?,XX,?
72	Standard risk	54,XY,+3,+5,+6,+9,+11,+15,+17,+19,del(1)(p)
73	Standard risk	54,XY,+3,+5,+7,+9,+11,+15,+19,+21
74	Standard risk	?,XY,t(9;14),+?
75	Standard risk	56,XY,+3,+4,+5,+6,+7,+9,+11,+15,+19,+21,dup(8)(q)
76	Standard risk	46,XX,t(11;14)
77	Standard risk	46,XX,dup(8)(q)
78	Standard risk	46,XY,t(11;14)
79	Standard risk	46,XY,t(11;14)
80	Standard risk	46,XX,t(11;14)
81	Standard risk	47,XY,t(11;14),+19
82	Standard risk	53,XY,+3,+5,+7,+9,+11,+15,+19
83	High risk	46,XX,dup(1)(q),dup(14)(q),del(16)(q)
84	High risk	45,XY,−13,dup(1)(q)
85	High risk	46,XY,t(4;14)
86	High risk	46,XY,del(17)(p)
87	High risk	46,XY,t(4;14),del(14)(q)
88	High risk	46,XX,del(17)(p)
89	High risk	46,XX,t(4;14)
90	High risk	?,XY,?
91	High risk	46,XX,t(4;14)
92	High risk	46,XX,t(4;14)
93	High risk	46,XX,t(11;14),dup(1)(q)
94	High risk	46,XX,del(8)(q),del(14)(q),del(17)(p)
95	High risk	47,XY,t(11;14),+11,dup(1)(q),dup(11)(q)
96	High risk	46,XY,dup(1)(q)
97	High risk	46,XY,t(11;14),dup(1)(q)
98	High risk	?,XY,?
99	High risk	46,XX,t(14;16)
100	High risk	46,XY,t(4;14)
101	High risk	?,XX,?
102	High risk	45,XY,−13,−Y,dup(1)(q)
103	High risk	45,XY,t(4;14),+3,−13,−18
104	High risk	50,XY,+3,+7,+9,+15,del(1)(p),dup(1)(q)
105	High risk	46,XY,t(4;14)
106	High risk	46,XX,t(4;14)
107	High risk	46,XY,del(1)(p),dup(1)(q)
108	High risk	57,XX,+3,+5,+7,+7,+9,+10,+15,+17,+18,+19,+19,dup(1)(q)
109	High risk	44,XX,t(11;14),−13,−X,dup(1)(q)
110	High risk	46,XX,t(4;14)
111	High risk	46,XY,t(11;14),dup(1)(q),del(13)(q)
112	High risk	51,XX,+3,+5,+7,+11,+19,dup(1)(q),del(13)(q)
113	High risk	46,XY,dup(4)(p),del(17)(p),del(20)(q)
114	High risk	51,XY,t(4;14),+3,+4,+15,+19,+21
115	High risk	46,XY,dup(1)(q)
116	High risk	46,XX,t(4;14),+8,−13
117	High risk	50,XX,t(4;14),+3,+5,+11,−13,+15,+19,del(1)(p)
118	High risk	49,XY,+3,+7,+9,−13,+15,dup(1)(q)
119	High risk	46,XY,t(4;14)
120	High risk	46,XY,dup(1)(q)
121	High risk	44,XY,−12,−14,+18,−Y,del(1)(p),dup(1)(q),del(2)(q),del(5)(p),dup(5)(q),del(6)(q), del(7)(p),dup(9)(q),del(11)(q),del(15)(q),del(16)(q),del(20)(p)
122	High risk	33,XY,−1,−2,−4,−6,−8,−10,−12,−13,−14,−16,−17,−20,−22,del(17)(p)
123	Ultra-high risk	44,XY,−13,−16,dup(1)(q),del(17)(p)
124	Ultra-high risk	53,XY,t(14;20),+3,+5,+7,+8,+11,−13,+18,+19,+21,dup(1)(q),dup(5)(p),dup(9)(q), dup(10)(q)
125	Ultra-high risk	45,XX,t(4;14),−13,dup(1)(q)
126	Ultra-high risk	45,XY,t(4;14),−13,del(1)(p),dup(1)(q)
127	Ultra-high risk	46,XX,t(4;14),del(1)(p),dup(1)(q)
128	Ultra-high risk	46,XX,t(14;16),dup(1)(q)
129	Ultra-high risk	46,XX,t(4;14),dup(1)(q)
130	Ultra-high risk	46,XY,t(4;14),dup(1)(q)
131	Ultra-high risk	42,XY,t(4;14),−2,−9,−13,−Y,del(1)(p),dup(1)(q),del(13)(p)
132	Ultra-high risk	45,XY,t(4;14),−13,dup(1)(q)
133	Ultra-high risk	56,XY,t(4;14),+2,+3,+5,+7,+9,+11,+15,+17,+19,+21,dup(1)(q)
134	Ultra-high risk	52,XY,t(11;14),+4,+7,+8,+9,+11,+15,del(17)(p)
135	Ultra-high risk	45,XY,t(4;14),−13,dup(1)(q)
136	Ultra-high risk	54,XY,+3,+5,+7,+11,+15,+15,+19,+19,dup(1)(q),del(17)(p)
137	Ultra-high risk	?,XX,+?,del(1)(p),dup(1)(q),del(17)(p)

**Table A3:** Course of treatment.

Parameter.	Total (*n* = 137)	Standard-Risk Genetics (*n* = 82)	High-Risk Genetics (*n* = 40)	Ultra-High-Risk Genetics (*n* = 15)	*p*-Value
First-line induction therapy received, n (%)	137 (100)	82 (100)	40 (100)	15 (100)	-
VRD	118 (86)	68 (83)	35 (87)	15 (100)	0.2036
Dara-VRD	5 (4)	3 (4)	2 (5)	0 (0)	-
VCD	8 (6)	6 (7)	2 (5)	0 (0)	-
VTD	1 (1)	1 (1)	0 (0)	0 (0)	-
VD	3 (2)	2 (2)	1 (3)	0 (0)	-
Dara-RD	2 (1)	2 (2)	0 (0)	0 (0)	-
Second-line induction therapy received, n (%)	5 (4)	4 (5)	1 (3)	0 (0)	-
Third-line induction therapy received, n (%)	3 (2)	3 (4)	0 (0)	0 (0)	-
Stem cell mobilisation ^a^, n (%)					
G-CSF only	41 (30)	24 (29)	12 (30)	5 (33)	0.9512
Chemotherapy ^b^, G-CSF	80 (58)	51 (62)	22 (55)	7 (47)	0.4662
Proteasome inhibitor ^c^, G-CSF	13 (10)	6 (8)	4 (10)	3 (20)	0.3024
HDCT					
Melphalan	43 (31)	24 (29)	14 (35)	5 (33)	0.8026
Bendamustin, Melphalan	29 (21)	18 (22)	7 (18)	4 (27)	0.7318
Treosulfan, Melphalan	65 (48)	40 (49)	19 (47)	6 (40)	0.8220
Tandem ASCT received, n (%)	14 (10)	5 (6)	7 (18)	2 (13)	0.1361
Maintenance therapy ^d^					
Lenalidomide	119 (87)	75 (92)	31 (78)	13 (87)	0.1006
Bortezomib	3 (2)	1 (1)	0 (0)	2 (13)	-
Carfilzomib	2 (2)	1 (1)	1 (3)	0 (0)	-
Ixazomib	3 (2)	0 (0)	2 (5)	1 (7)	-
Daratumumab	6 (4)	3 (4)	2 (5)	1 (7)	-
Pomalidomide, Elotuzumab	1 (1)	1 (1)	0 (0)	0 (0)	-
None	12 (9)	5 (6)	6 (15)	1 (7)	0.2518

VRD: bortezomib, lenalidomide, dexamethasone; Dara-VRD: daratumumab, bortezomib, lenalidomide, dexamethasone; VCD: bortezomib, cyclophosphamide, dexamethasone; VTD: bortezomib, thalidomide, dexamethasone; VD: bortezomib, dexamethasone; Dara-RD: daratumumab, lenalidomide, dexamethasone; HDCT: high-dose chemotherapy; ASCT: autologous stem cell transplantation; ^a^ data missing for 3 patients; ^b^ using vinorelbine or gemcitabine; ^c^ using ixazomib; ^d^ some patients received several drugs in combination.
